# Phytochemical, Phytotherapeutical and Pharmacological Study of *Momordica dioica*

**DOI:** 10.1155/2014/806082

**Published:** 2014-08-12

**Authors:** Sattya Narayan Talukdar, Mohammad Nazir Hossain

**Affiliations:** ^1^Biomedical Research Unit, Biochemistry Department, Primeasia University, Banani, Dhaka-1213, Bangladesh; ^2^School of Science, Primeasia University, Banani, Dhaka-1213, Bangladesh

## Abstract

Momordica dioica is a perennial, dioecious, cucurbitaceous climbing creeper (commonly known as kakrol, spiny gourd or teasle gourd). It is native to Asia with extensive distribution in India and Bangladesh. It is used not only as preventive and curative agent for various diseases but also as vegetable with a significant nutritional value over thousands of years. This review aims to take an attempt to evaluate the phytochemical, ethnobotanical, phytotherapeutical and pharmacological properties of kakrol according to the view of traditional medicinal plant based treatment including ayurveda along with recent scientific observations. Kakrol is considered as an underutilized vegetable, although having significant presence of certain compounds containing higher nutritional value than many frequently consumed vegetables. Moreover, as a traditional medicinal plant, it is still potential for its phytochemical components that increase the demand of further extensive evaluation to justify its other therapeutical roles. Therefore, this effort will be helpful to researchers who interested to disclose the unjustified phytotherapeutical role of Momordica dioica.

## 1. Introduction

**Figure 1:**
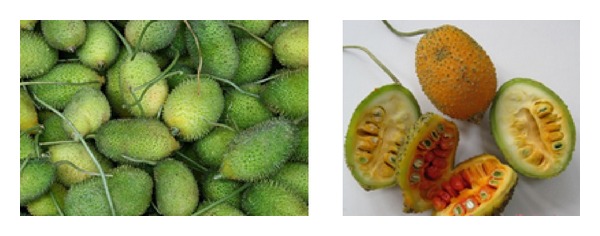
*Momordica dioica* (Photo credit: https://www.flickr.com/, http://www.tropicalfruitandveg.com/).

*Momordica dioica* Roxb. is a perennial, dioecious (2*n* = 28) climber included in Cucurbitaceae family (Figure 1). Momordica genus contains about 80 species [1, 2]. According to the latest revision of Indian* Momordica*, there are six well identified species of which four are dioecious and two are monoecious [3]. Although this genus is originated from Indo-Malayan region, it is now found to grow in India, Bangladesh, Srilanka, Myanmar, China, Japan, South East Asia, Polynesia, Tropical Africa, and South America [4, 5]. Its cultivation up to an altitude of 1500 meters in Assam and Garo hills of Meghalaya is reported [6]. It is commonly known as spine gourd, teasel gourd or small bitter gourd worldwide whereas in Bangladesh it is known as kakrol and in India as kankro, kartoli, kantoli, kantola, kantroli, ban karola, or janglee karela [7–10]. Kakrol is about 5–7 meters in length, a popular summer vegetable of which its fruit, young twigs and leaves are used as vegetable [11–13].

## 2. Phytochemical and Nutrient Study

The fruit of* Momordica dioica* contains ashes: 9.1%, crude protein: 5.44%, crude lipid: 3.25%, crude fiber: 22.9%, and carbohydrate: 59.31%. Its fruit has high energy value (288.25 kcal/100 g) in dry weight. Its mineral ranges (mg/100 g dry weight,) are: potassium (4.63), sodium (1.62), calcium (7.37), iron (5.04), and zinc (3.83) [14]. In another investigation, its nutritional value of per 100 g edible fruit is reported to contain 84.1% moisture, 7.7 g carbohydrate, 3.1 g protein, 3.1 g fat, 3.0 g fiber and 1.1 g minerals and small quantities of essential vitamins like carotene, thiamin, riboflavin and niacin [15].

Ali and Deokule evaluated some of its micronutrient and secondary metabolites as follows: calcium: 0.5 mg/g, sodium: 1.5 mg/g, potassium: 8.3 mg/g, iron: 0.14 mg/g, zinc: 1.34 mg/g, protein: 19.38%, fat: 4.7%, total phenolic compound: 3.7 mg/g, phytic acid: 2.8 mg/g, and ash value: 6.7% [16]. Moreover, its fruit is recommended as nutritionally rich source of protein and good source of lipid, crude fiber, carbohydrate, iron, calcium, phosphorous. Additionally, it is the highest amount of carotene (162 mg/100 g of edible portion) container amongst the cucurbitaceous vegetables [17–19]. The ash content is reported as 3-4% containing a trace of manganese [20].

Tirmizi et al. screened it as a potential source of chromium and zinc [21]. Whereas,* Momordica dioica* (peeled) contained 0.27 mg/kg of chromium and 4.91 mg/kg of zinc,* Momordica dioica* (unpeeled) contained 0.26 mg/kg of chromium and 11.0 mg/kg of zinc. The protein content of leaves and dry weight of aerial plant parts remained higher in male as compared to female defruited and monoecious plants [22]. The fruit contains higher amount of ascorbic acid and iodine [23, 24]. The presence of secondary metabolites of fruit including alkaloids, steroids, triterpenoids, and saponins was determined [25]. Among them, four compounds were isolated from ethyl acetate extract and five compounds were isolated from methanol extract consisting of alkaloids and flavonoids with NH and C=O functional groups, respectively. The alkaloids present in seed and root were called momordicin and* Momordica foetida*, respectively [26]. Phytochemical investigations summurized in Table 1 also showed the presence of lectins, *β*-sitosterol, saponin glycosides, triterpenes of ursolic acid, hederagenin, oleanolic acid, *α*-spinasterol, stearic acid, gypsogenin, momodicaursenol, and three new compounds named 3*β*-o-benzoyl-11-oxo-ursolic acid, 3*β*-o-benzoyl-6-oxo-ursolic acid, and 3-o-*β*-D-glucuronopyranosyl gypsogenin [27–32].

## 3. Ethnobotanical and Phytotherapeutical Study

According to Ayurveda (Table 2), not only its fruits have diuretic, laxative, hepatoprotective, antivenomous, antihypertensive, anti-inflammatory, antiasthmatic, antipyretic, antileprosy, antidiabetic, and antidepressant properties but also its leaves have antihelminthic, aphrodisiac, antihemorroidal, hepatoprotective, antibronchitic, antipyretic, antiasthmatic, and analgesic properties [33, 34]. Fresh fruit juice and cooked fruit in small amount of oil are prescribed for hypertension and diabetes, respectively. Oral administration of 50 mL of root juice is advised once a day with empty stomach to beat diabetes. The juice of root is a domestic remedy for the inflammation caused by contact with the urine of the house lizard. The juice of the leaves are mixed with coconut, pepper, red sandalwood, and so forth in order to form an ointment and applied to the head to relieve pain. Dried fruit powder applied into the nostrils produces a powerful errhine effect and provokes a copious discharge from the schneiderian mucous membrane [35]. Root juice has stimulant, astringent, antiseptic, antidiabetic, anti-inflammatory, and antiulcerant effect. The mucilaginous tubers act as antihelminthic, spermicidal, and antifertility abortifacient agent [36]. The root of the male plant is used in snake bites and scorpion sting [37]. The superficial use of root paste over the whole body is believed to act as a sedative in high fever with delirium [38, 39]. Beside the superficial and oral administration of leaf paste for skin disease, tender fruits are rubbed on skin for pimples and acne and roasted seeds are used for eczema and other skin problems [40]. Root powder is also applied for softening skin and reducing perspiration. The protective role of the leaves against chronic skin diseases is also reported. A preparation called “Panchatikta ghrita” is made by boiling 800 g each of neem bark, leaves of* Momordica dioica*,* Solanum surattense*,* Tinospora cordifolia,* and bark of* Adhatoda vasica*, in 5-6 liters of water up to its reduction to quarter and then adding of 3.5 liters of butter and about 3 kg myrobalans and is recommended as one tablespoonful with little hot milk internally twice daily in chronic skin diseases [41]. Mucilaginous tuber of female plant and toasted root are used in bleeding piles and bowel infections. The traditional use of* Momrdica dioica* against bleeding piles (hemorrhoids) is also reported [42, 43].

## 4. Pharmacological Study

### 4.1. Antioxidant Activity

Compounds derived from natural sources are capable of providing protection against free radicals [44]. The alcoholic extract inhibited the formation of oxygen derived free radicals (ODFR)* in vitro* with 4000 *μ*g/mL ascorbic system [45]. In another work, the free radical scavenging potential of the tuberous roots was studied by different* in vitro* methods, namely, DPPH radical scavenging, ABTS radical scavenging, iron chelating activity, total antioxidant capacity, and haemoglobin glycosylation assay. Total antioxidant capacity of ethanolic extract was found to be 26 *μ*g/mL which is equivalent to ascorbic acid. Moreover, its ethanol extract showed percentage inhibition of haemoglobin glycosylation as 66.63 and 74.14 at conc. of 500 and 1000 *μ*g/mL, respectively, while that of standard DL *α*-tocopherol was 61.53% and 86.68% inhibition at same concentration [46]. The antioxidant activities of methanol and aqueous extract of fruits were analyzed and the presence of phenolic compounds, flavonoids, sterol, alkaloids, amino acids, and so forth, were found [47]. Among those compounds, due to the presence of flavonoids, its fruit was reported as a potent antioxidant [48].

### 4.2. Analgesic Activity

Ilango et al. and Vaidya and Shreedhara reported that both hexane extract and soluble portion of methanolic extract of* Momordica dioica* fruit pulp exhibited analgesic activity when compared to standard drug [49, 50]. Petroleum ether, ethyl acetate, and methanol extracts exhibited significant analgesic activity in acetic acid induced writhing syndrome when compared to the vehicle treated control group. But among them petroleum ether and methanol extract gave more significant analgesic activity than ethyl acetate extract [51].

### 4.3. Nephroprotective Activity

The ethanol extract of seeds was screened and marked nephroprotective as well as curative activities was found without any toxicity caused by nephrotoxin-like gentamicin [52]. The nephroprotective and curative activities of its fruit extract ware also observed [53]. Gupta et al. evaluated the renal protective effect of* Momordica dioica* extract in streptozotocin-diabetic rats [54].

### 4.4. Neuroprotective Activity

The effect of methanol and aqueous extract of fruit pulp was observed on the central nervous system by using neuropharmacological experimental models in mice. These extracts were used for a dose-dependent reduction of the onset and duration of a reduction in locomotor activity. It was suggested that methanol and aqueous extract of fruit pulp (100 mg/kg and 200 mg/kg) had neuroprotective activities [55].

### 4.5. Antiallergic Activities

The antiallergic activity of its extract in mice was observed [56]. The alcoholic extract was evaluated and its efficacy to inhibit passive cutaneous anaphylaxis was found in mouse and rat [57].

### 4.6. Antiulcer Activity

Vijayakumar screened* Momordica dioica* extract mediated antiulcerogenic effect on ethanol-induced ulcer model of rat. A significant decrease occurred in the level of H^+^-K^+^ATPase, volume of gastric juice, and acid output. Gastric wall mucus, p^H^, and catalase enzyme were increased significantly but antioxidant enzyme levels of superoxide dismutase were decreased [58]. Its gastroprotective and ulcer healing activities were also observed by Vijayakumar et al. [59].

### 4.7. Anticancer Activity

Luo et al. showed that the CHCl_3_ extract of roots and five isolated constituents had anticancer activity during pharmacological testing on cancer cell (L1210). The growth inhibitory index (%) of *α*-spinasterol-3-o-*β*-D-glucopyranoside was shown to be 50%, at the dose of 4 *μ*g/mL [31].

### 4.8. Antimicrobial Activity

Shrinivas et al. studied methanolic extract and aqueous extract of fruit and found that methanolic extract had more promising antimicrobial activity [47]. Arekar et al. screened antibacterial activities of ethyl acetate extract. The concentration of 200 *μ*g/disc was more active against* E. coli* compared to* S. aureus, S. paratyphi*, and* P. mirabilis* bacteria. Ethyl Acetate extract of* in vitro* shoot culture (yield: 0.26%) showed maximum inhibition zone against* S. paratyphi* and* P. mirabilis* while ethyl acetate extract of* in vitro* callus culture (yield: 21.5%) showed maximum inhibition zone against* S. aureus* [60]. On the other hand, Singh et al. found its no promising antimycobacterial activity [61].

### 4.9. Antidiabetic Activity

Antidiabetic specifically oral hypoglycemic effects of* Momordica dioica* in rat model was screened by Fernandopulle et al. [62]. Reddy et al. and Singh et al. showed aqueous, chloroform, ethyl acetate and ethanolic extract of fruit mediated antidiabetic activity in alloxan induced experimental rats [63, 64]. Moreover, Sharma and Arya reported ethyl acetate and ethanol extract containing steroids, triterpenoids had potential role in alloxan-induced diabetic rats and broadly type 2 diabetes [65]. Gupta et al. investigated the antidiabetic and renal protective effect of* Momordica dioica* methanolic extract (MDMtE) in streptozotocin-treated diabetic rats. MDMtE treatment markedly reduced serum glucose and increased serum insulin and urea levels. Furthermore, histologic observation of kidney of diabetic rats showed degenerative changes in glomerulus and renal tubules [54].

### 4.10. Antimalarial Activity

Misra et al. screened alcoholic extract* in vivo* and* in vitro* for antimalarial effect against NK65 strain of* Plasmodium berghei*,* Jurinea macrocephala*, and* Aegle marmelos* and found them to possess schizontocidal activity [66].

### 4.11. Anti-Inflammatory Activity

The anti-inflammatory effect of the alcoholic extract of roots was evaluated during CCl_4_ induced hepatotoxicity [45]. Ilango et al. evaluated both hexane extract and methanolic extract of fruit pulp mediated anti-inflammatory activities [49].

### 4.12. Hepatoprotective and Antihepatotoxic Activity

CCl_4_ induced hepatotoxicity prevention of methanol extract of* Momordica dioica* was observed by Chaudhary et al. [67]. Although Govind reported the hepatoprotective and antihepatotoxicity effect of leaf, Kumar et al. specifically mentioned the role of aqueous and methanol extract of leaves against it [68, 69]. Jain et al. examined leaf as a potent hepatoprotective agent against CCl_4_ induced hepatic damage in rats by* in vivo* antioxidant and free radical scavenging activities. They were positive for both ethanolic and aqueous extracts although ethanolic extract was found more potent hepatoprotective [48]. Kushwaha et al. evaluated the flavonoidal fraction from ethanolic extract of fruit mediated hepatoprotective activity in wistar strain of albino rats of either sex against CCl_4_ induced hepatic damage [70]. Rakh et al. reported that the alcoholic extract of roots significantly reduced CCl_4_ induced hepatotoxicity in rats by inhibiting the formation of radicals* in vitro* [56]. The saponin fraction of* Momordica dioica* (27.5 and 55 mg/kg) administered to the CCl_4_ treated rats to protect the liver cells from liver damages on hepatocytes and silymarin (100 mg/kg), a well-known natural antihepatotoxic drug was used as standard [71]. The hexane extract and ethyl acetate soluble fraction of the methanolic extract of the fruit pulp at a dose of 400 mg/kg administered for 7 days in rat exhibited a significant therapeutic effect [72]. Sato et al. observed significant lowering of liver cholesterol and triacylglycerol levels in rats. Fecal lipid excretion was increased and lymphatic transport of triacylglycerol and phospholipids were decreased in rats which were fed the kakrol after permanent lymph cannulation. Moreover, *n*-butanol extract caused a significant reduction in the pancreatic lipase activity* in vitro* and liver lipids by inhibiting lipid absorption [73].

### 4.13. Antifertility Activity

Shreedhar et al. reported the antifertility activity of ethanolic and aqueous extract of* Momordica dioica* root. The extracts showed moderate estrogenic activity and caused significant increase in uterine weight. Moreover, at a dose of 200 mg/kg, aqueous extract showed 83% and ethanolic extract showed 100% abortifacient activity [74]. Kudaravalli evaluated the ethanolic extract of fruit mediated antifertility activities of female rats but found no male antifertility activity at the dose of 250 mg/kg [75].

### 4.14. Antiedemic Activity

Shreedhara and Vaidya administered the alcoholic extract orally which significantly reduced carrageenan-induced paw edema. The activity was compared with ibuprofen (200 mg/kg) [45].

### 4.15. Antifeedant, Insecticidal, Grain Protectant, and Allelopathic Activity

Mishra et al. reported the role of* Momordica dioica* seed oil as insecticide and found satisfactory level of natural insecticidal activity up to 100% mortality at 4% conc. in 24 hours. Moreover, its lower conc. up to 2% was found to be effective but for 100% mortality longer time was required. They suggested the presence of alkaloid momordicin in oil was responsible for it [76]. In another work, Mishra et al. evaluated its seed oil's potential as grain protectant against* Callosobruchus chinensis* upon the stored legume-pulse grain. It was applied as a dose of 6–8 mL/kg to legume pulse grain sample for 60 days. As a result, appeared degree of dehusking was increased (%) from 40.00 to 72.59, 59.88 to 92.44, 63.39 to 87.50 and 57.00 to 79.43 for Pigeon pea (*Canjanas cajan*), Chickpea (*Cicer arietinum*), Urdbean (*Phaseolus mungo*), Mungbean (*Phaseolus radiatus*), respectively [77]. Narasimhan et al. and Meriga et al. reported that the hexane extract and ethyl acetate extract of the fruit pulp had moderate and concentration dependent antifeedant activity against* Spodoptera litura* [78, 79]. Allelopathy refers to the chemical inhibition of one species by another by releasing chemicals into the environment where it affects the development and growth of neighboring plants. Ahire and Deokule observed the leaf extract of* M. dioica* mediated allelopathic activity on seedling growth as well as seed germination of* P. aconitifolius* and found major toxicity at a dose of 2.0% and 2.5% w/v of phytoextracts [80]. These above information are summarized in Table 3.

## 5. Conclusion

The traditional use of medicinal plants has a long history. Ancient people as well as our ancestors were mainly dependent on plants for their recovery against diseases. But, the recent tendency to avoid natural sources rather than artificial sources against disease is frustrating. Because continuous reports of antibiotic resistance as well as the side effects of synthetic drugs all over the world are indicating a global health alert. The higher occurrence rate of worldwide diabetes, cancer, obesity, hypertension, and neurodegenerative diseases becomes alarming to all. Huge researches are carried out to find the causes and remedies of them. Therefore, to search for a better alternative than synthetic drug becomes the demand of time.

Medicinal plants may be a good option to play pivotal role against such complications. But, before that their previous use and curability should be justified. Medicinal plants are the source of enormous secondary metabolites. The diverse role of secondary metabolites may provide a key of the door of undiscovered remedy against diseases. In that case, long term research on medicinal plant is essential to justify their potential. Moreover, the use of medicinal plants is important for its ecofriendly significance as well as its fewer side effects than other synthetic drugs. Additionally, it will be comparatively safer and cheaper than man-made drugs formulation.

South Asia, as one of the highest sources of medicinal plant in the world, provides enormous medicinal plants including kakrol, having several significant folk uses but not clinically evaluated till now. Therefore, vast chances have been created to justify the dynamic ethnobotanical and phytotherapeutical roles of several plants for future researchers. This paper has mainly focused on the phytotherapeutical and pharmacological potential of* Momordica dioica*. As it contains significant amount of antioxidant, vitamin, secondary metabolites, and other important ingredients, these may be helpful to fight against several diseases including diabetes, cancer, and neurodegenerative diseases. For example, ethyl acetate and ethanol extract of kakrol containing steroids, tritepenoids etc. have potential role in alloxan-induced diabetic rats and broadly type 2 diabetes. Similarly, methanol and aqueous extract of its fruit pulp have neuroprotective activities.

Therefore, this paper will be fruitful if it stimulates the researcher's emphasis to justify the unrevealed but potential therapeutic properties of* Momordica dioica* against diabetes, cancer, neurodegenerative disease, and other life threatening disorders.

## Figures and Tables

**Table 1:** Nutrient and phytochemical study of *Momordica dioica* as described in this paper.

Plant part	Classification	Compound	Extract or preparation	Reference
Fruit	Crude protein	—	Quantitative analysis showed 5.44%	[14]
	Protein	—	Quantitative analysis showed 3.1/100 g	[15]
		—	Quantitative analysis showed 19.38%	[16]
	Crude lipid	—	Quantitative analysis showed 3.25%	[14]
	Fat	—	Quantitative analysis showed 3.1/100 g	[15]
		—	Quantitative analysis showed 4.7%	[16]
	Crude fiber	—	Quantitative analysis showed 22.9%	[14]
	Carbohydrate	—	Quantitative analysis showed 59.31%	[14]
		—	Quantitative analysis showed 7.7/100 g	[15]
	Niacin	—	Not specified	[15]
	Thiamin	—	Not specified	[15]
	Carotene	—	Not specified	[15]
		—	Quantitative analysis showed 162 mg/100 g of edible portion	[18, 19]
	Ascorbic acid	—	Not specified	[24]
	Potassium	—	Quantitative analysis showed 4.63 mg/100 g dry weight	[14]
		—	Quantitative analysis showed 8.3 mg/g	[16]
	Sodium	—	Quantitative analysis showed 1.62 mg/100 g dry weight	[14]
		—	Quantitative analysis showed 1.5 mg/g	[16]
	Calcium	—	Quantitative analysis showed 7.37 mg/100 g dry weight	[14]
		—	Quantitative analysis showed 0.5 mg/g	[16]
	Iron	—	Quantitative analysis showed 5.04 mg/100 g dry weight	[14]
		—	Quantitative analysis showed 0.14 mg/g	[16]
	Zinc	—	Quantitative analysis showed 3.83 mg/100 g dry weight	[14]
		—	Quantitative analysis showed 1.34 mg/g	[16]
		—	Not specified	[21]
		—	Quantitative analysis showed 4.91 mg/kg (peeled), 11.0 mg/g (unpeeled)	[22]
	Manganese	—	Not specified	[20]
	Iodine	—	Not specified	[23]
	Chromium	—	Quantitative analysis showed 0.27 mg/kg (peeled), 0.26 mg/kg (unpeeled)	[22]
		—	Not specified	[21]
	Phytic acid	—	Quantitative analysis showed 2.8 mg/g	[16]
	Total phenolic compound	—	Quantitative analysis showed 3.7 mg/g	[16]
	Alkaloids	—	Identified in ethyl acetate, methanol extract	[25]
	Flavonoid	—	Identified in methanol, hexane extract	[25]
	Steroids	—	Identified in ethyl acetate, methanol, aqueous extract	[25]
	Saponins	—	Identified in methanol, aqueous extract	[25]
	Triterpenoids	—	Identified in ethyl acetate, methanol, aqueous extract	[25]

Seed	Alkaloid	Momordicin	Identified in seed oil	[26]
	Lectin	Anti-H-Lectin	Not specified	[30]

Root	Alkaloid	*Momordicafoetida *	Not specified	[26]
	Stearic acid	—	Identified in methanol extract	[31]
	Steroid	*α*-spinasterol octadecanoate	Identified in methanol extract	[31]
		*α*-spinasterol-3-O-*β*-D-glucopyranoside	Identified in methanol extract	[31]
	Triterpenoid	Oleanolic acid	Identified in methanol extract	[32]
		Gypsogenin	Identified in methanol extract	[32]
		Hederagenin	Identified in methanol extract	[32]
		3*β*-O-benzoyl-6-oxo-ursolic acid	Identified in methanol extract	[32]
		3*β*-O-benzoyl-11-oxo-ursolic acid	Identified in methanol extract	[32]
		3-O-*β*-D-glucopyranosyl hederagenin	Identified in methanol extract	[31]
		3-O-*β*-D-glucopyranosyl gypsogenin	Identified in methanol extract	[31]
		3-O-*β*-D-glucuronopyranosyl gypsogenin	Identified in methanol extract	[31]

**Table 2:** Ethnobotanical use of *Momordica dioica* as described in this paper.

Plant's part	Ethnobotanical use	Preparation or Mode of use	Reference
Fruit	Hypertension	Fresh fruit juice	[35]
	Diabetes	Cooked fruit in small amount of oil	[35]
	Pimple and acne protectant	Tender fruits are rubbed on skin for pimples and acne	[40]
	Diuretic	Not specified	[33, 34]
	Laxative	Not specified	[33, 34]
	Hepatoprotective agent	Not specified	[33, 34]
	Antihypertensive	Not specified	[33, 34]
	Anti-inflammatory agent	Not specified	[33, 34]
	Antipyretic	Not specified	[33, 34]
	Antivenomous agent	Not specified	[33, 34]
	Antiasthmatic agent	Not specified	[33, 34]
	Antidepressant	Not specified	[33, 34]
	Antileprosy agent	Not specified	[33, 34]

Root	Diabetes	Oral administration of 50 mL of root juice is advised once a day with empty stomach.	[35, 36]
	Anti-inflammatory agent	The juice of the root is a domestic remedy for the inflammation caused by contact with the urine of the house lizard.	[35, 36]
	Stimulant	Root juice	[36]
	Antiseptic	Root juice	[36]
	Antiulcerant	Root juice	[36]
	Antitoxic agent	The root of the male plant uses in snake bites and scorpion sting	[37]
	Antipyretic	The root paste smearing over the whole body act as a sedative fever with delirium	[38, 39]
	Skin softening agent	Root powder is applied for softening skin	[41]
	Antiperspirant	Root powder is applied for reducing perspiration.	[41]
	Antihemorroidal agent	Toasted roots are used in bleeding piles	[42, 43]
	Bowel infection reducer	Toasted roots are used in bowel infections	[42]

Mucilaginous tuber	Antihelminthic agent	Not specified	[36]
	Spermicidal agent	Not specified	[36]
	Antifertility agent	Not specified	[36]
	Antihemorroidal agent	Mucilaginous tuber of female plant are used in bleeding piles	[42, 43]
	Bowel infection reducer	Mucilaginous tuber of female plant are used in bowel infections	[42]

Seed	Eczema protectant	Roasted seeds are used for eczema and other skin problems	[40]

Leaf	Analgesic	Leaf juice is mixed with coconut, pepper, red sandalwood, and so forth in order to form an ointment to relieve pain.	[35]
	Antihelminthic	Not specified	[33, 34]
	Antihemorroidal	Not specified	[33, 34]
	Antibronchitic	Not specified	[33, 34]
	Skin disease reducer	A preparation called “Panchatikta ghrita” is made by boiling 800 g each of neem bark, leaves of *Momordica dioica, Solanum surattense, Tinospora cordifolia*, and bark of *Adhatoda vasica*, in 5-6 liters of water up to its reduction to quarter and then the addition of 3.5 liters of butter and 3 kg myrobalans, is recommended as one tablespoonful with little hot milk internally twice daily in chronic skin diseases	[40, 41]

**Table 3:** Pharmacological evaluation of *Momordica dioica *described in the paper.

Pharmacological activity	Part of plant	Extract/preparation	Detail effect	Reference
Antioxidant activity	Root	Alcoholic extract	Inhibited the formation of oxygen derived free radicals (ODFR) *in vitro* with 4000 *μ*g/mL ascorbic system.	[45]
	Root	Ethanol extract	DPPH radical scavenging, ABTS radical scavenging, iron chelating activity, total antioxidant capacity and haemoglobin glycosylation assay were studied. Total antioxidant capacity was 26 *µ*g/mL equivalents to ascorbic acid.	[46]
	Fruit	Methanol, aqueous extract	Found the presence of phenolic compound, flavonoids, sterol, alkaloids and amino acids.	[47]
	Leaf	Ethanol, aqueous extracts	The presence of flavonoids was reported as a potent antioxidant	[48]

Analgesic activity	Fruit	Hexane, methanol extract	Exhibited analgesic activity when compared to standard drug	[49]
	Fruit	Petroleum ether, methanol, ethyl acetate extract	Petroleum ether and methanol extract gave more significant analgesic activity than ethyl acetate extract.	[51]

Nephroprotective activity	Seed	Ethanol extract	Found marked nephroprotective and curative activities without any toxicity caused by nephrotoxin-like gentamicin.	[52]
	Fruit	Ethanol extract	Observed significant reduction in GSH and an increase in malondialdehyde (MDA) production.	[53]

Neuroprotective activity	Fruit	Methanol and aqueous extract	Methanol and aqueous extract of fruit pulp (100 mg/kg and 200 mg/kg) had neuroprotective activities.	[55]

Antiallergic activities	Seed	Alcoholic extract	The antiallergic activity of extract in mice was observed.	[56]
	Not specified	Alcoholic extract	Found its efficacy to inhibit passive cutaneous anaphylaxis in mouse and rat.	[57]

Antiulcer activity	Fruit	Ethanol extract	Decreased the level of H^+^-K^+^ATPase, volume of gastric juice, and acid output. Gastric wall mucus, p^H^ and catalase enzyme were increased significantly. Antioxidant enzyme levels of superoxide dismutase were decreased.	[58]
	Fruit	Hydro alcohol extract	Gastroprotective and ulcer healing activities were observed.	[59]

Anticancer activity	Root	Methanol extract	The growth inhibitory index (%) of *α*-spinasterol-3-o-*β*-D-glucopyranoside was shown to be 50%, at the dose of 4 *µ*g/mL while testing on cancer cell (L1210).	[31]

Antimicrobial activity	Fruit	Methanol, aqueous extract	Found methanolic extract had more promising antimicrobial activity.	[47]
	Root, Leaf	Ethyl acetate extract	The concentration of 200 *μ*g/disc was more active against *E. coli* compared to, *S. paratyphi*, and *P. mirabilis *bacteria.	[60]

Antidiabetic activity	Fruit	Aqueous extract	Oral hypoglycemic effect of *Momordica dioica* in rat model was screened.	[62]
	Fruit	Chloroform, ethyl acetate, and alcohol extract	Ethyl acetate and ethanol showed significant antidiabetic activity at a dose of 200 mg/kg.	[63]
	Fruit	Aqueous, hexane, chloroform, and ethanol extract	Aqueous extract showed maximum fall (52.8%) in 0 to 1 h fasting blood glucose in glucose tolerance test compared to hexane (39%), chloroform (37.2%), and ethanol (37.7%) extract in normal healthy rats.	[64]
	Not specified	Ethyl acetate and ethanol extract	Screened potential role in alloxan-induced diabetic rats and broadly type 2 diabetes.	[65]
	Fruit	Methanol extract	Markedly reduced serum glucose and increased serum insulin and urea levels.	[54]

Antimalarial activity	Not specified	Alcoholic extract	Misra screened extract *in vivo* and *in vitro* against NK65 strain of *Plasmodium berghei*, *Jurinea macrocephala*, *Aegle marmelos* and found to possess schizontocidal activity.	[66]

Anti-inflammatory activity	Root	Alcoholic extract	Significantly reduced carrageenan-induced paw edema when administered orally (200 mg/kg) and the activity was comparable with ibuprofen (200 mg/kg, p.o.)	[45]
	Fruit	Hexane, methanol extract.	Both extracts exhibited anti-inflammatory activities when compared to standard drug	[49]

Hepatoprotective and antihepatotoxic activity	Root	Ethanol extract	Prevented CCl_4 _induced hepatotoxicity at a dose of 200 mg/kg	[67]
	Leaf	Aqueous, methanol extract	Reported hepatoprotective and antihepatotoxicity effect of leaf.	[68, 69]
	Fruit	Ethanol extract	*Evaluated* hepatoprotective activity in wistar strain of albino rats of either sex against CCl_4_ induced hepatic damage.	[70]
	Leaf	Ethanol, aqueous extracts	Ethanol extract was found more potent hepatoprotective against CCl_4 _induced hepatic damage in rats by *in vivo* free radical scavenging activities.	[48]
	Root	Alcohol extract	Reduced CCl_4_ induced hepatotoxicity in rats by inhibiting the formation of radicals *in vitro* with ascorbic system.	[56]
	Fruit	Methanol extract	The saponin fraction of *Momordica dioica* (27.5 and 55 mg/kg) administered to the CCl_4_ treated rats to protect the liver cells from liver damages on hepatocytes and silymarin (100 mg/kg).	[71]
	Fruit	Methanol extract	Exhibited a significant therapeutic effect at a dose of 400 mg/kg administered for 7 days in rat.	[72]
	Fruit	n-butanol extract	Observed significant lowering of liver cholesterol and triacylglycerol levels in rats. Moreover, n-butanol extract caused a significant reduction in the pancreatic lipase activity *in vitro*.	[73]

Antifertility activity	Root	Ethanol, aqueous extract	Found moderate estrogenic activity including significant increase in uterine weight and abortifacient activity.	[74]
	Fruit	Ethanolic extract	Found antifertility activities of female rats but no male antifertility activity at the dose of 250 mg/kg	[75]

Antiedemic activity	Root	Alcoholic extract	Showed significant reduction of carrageenan-induced paw edema.	[45]

Insecticidal activity	Seed	Seed oil	Alkaloid momordicin in seed oil was responsible for 100% mortality at 4% conc. in 24 hours.	[76]

Grain protectant activity	Seed	Seed oil	Seed oil was grain protectant against *Callosobruchus chinensis *	[77]

Antifeedant activity	Fruit	Hexane and ethyl acetate extract	Showed antifeedant activity against *Spodoptera litura *	[78, 79]

Allelopathic activity	Leaf	Aqueous extract	Leaf extract has allelopathic activity on seedling growth and seed germination of *P. aconitifolius *	[80]
